# Utilization of Waste Materials for Microbial Carrier in Wastewater Treatment

**DOI:** 10.1155/2016/6957358

**Published:** 2016-07-20

**Authors:** H. T. Le, N. Jantarat, W. Khanitchaidecha, K. Ratananikom, A. Nakaruk

**Affiliations:** ^1^Department of Civil Engineering, Faculty of Engineering, Naresuan University, 99 Moo 9, Tapho Subdistrict, Muang District, Phitsanulok 65000, Thailand; ^2^Centre of Excellence for Innovation and Technology for Water Treatment, Naresuan University, 99 Moo 9, Tapho Subdistrict, Muang District, Phitsanulok 65000, Thailand; ^3^Department of Science and Mathematics, Faculty of Agro-Industrial Technology, Kalasin University, 62/1 Kaset Sombun Road, Kalasin Subdistrict, Muang District, Kalasin 46000, Thailand; ^4^Department of Industrial Engineering, Faculty of Engineering, Naresuan University, 99 Moo 9, Tapho Subdistrict, Muang District, Phitsanulok 65000, Thailand

## Abstract

This research focused on the ammonium-nitrogen (NH_4_-N) removal from the domestic wastewater using the attached growth reactors. Two types of waste material of corncob (biodegradable material) and concrete (nonbiodegradable material) were used as the carrier for microorganisms' attachment. During operation, both reactors achieved absolutely high performance of ammonium removal (up to 99%) and total nitrogen removal (up to 95%). The significant advantage of corncob carrier was that the corncob was able to be a source of carbon for biological denitrification, leading to no external carbon requirement for operating the system. However, the corncob caused an increasing turbidity of the effluent. On the other hand, the concrete carrier required the minimal external carbon of 3.5 C/N ratio to reach the good performance. Moreover, a longer period for microorganisms' adaptation was found in the concrete carrier rather than the corncob carrier. Further, the same physiological and biochemical characteristics of active bacteria were found at the two carriers, which were negative gram, cocci shape, and smooth and white-turbid colony. Due to the effluent quality, the concrete was more appropriate carrier than the corncob for wastewater treatment.

## 1. Introduction

Domestic wastewater contains high concentration of nitrogen which is from products through household consumption and human metabolism. When the large volume of domestic wastewater is discharged to the natural water resource, it can cause the environmental problem of eutrophication or algae bloom. The consequences are depletion of dissolved oxygen, water quality degradation, death of aquatic lives, and odor and taste problems. The enormous damage to ecological, health, and economic values from the eutrophication has become serious issues in recent decades such as swimming bans in De Kuil Lake in Netherlands [1], less abundant aquatic lives in the Gulf of Mexico [2], and smell of drinking water in Changjiang Estuary [3].

Recently, various biological technologies have been proposed for efficient nitrogen removal from wastewater including simultaneous nitrification and denitrification: SND [4], anammox-based reactor [5], membrane bioreactor [6], and combination of activated carbon and short-cut nitrification [7]. Among the above mentions, the SND is a widely used technology for domestic wastewater plant, due to its simplicity and ease of operation. There are two major mechanisms and two microorganism types; the ammonium-nitrogen (NH_4_-N) is converted to nitrite-nitrogen (NO_2_-N) and continued to nitrate-nitrogen (NO_3_-N) by autotroph (named nitrification process), and later NO_3_-N is reduced to nitrogen gas (N_2_) by heterotroph (named denitrification process). Since the organic carbon contained in the domestic wastewater was low and insufficient for complete denitrification, the external carbon including acetate and ethanol is necessary for the SND system. However, an overdose of external carbon can cause another contamination of organic carbon which presents in the parameter of biological oxygen demand (BOD), and the BOD contamination requires further treatment. In addition, the traditional SND of suspended sludge has some drawbacks including long lag phase for biomass adaptation, washout of biomass, and sensitive system to pH and loading [8].

An attached growth reactor has been proposed to overcome the drawbacks of the traditional SND system. In the attached growth reactor, the carrier is used for biomass attachment and resulted in high active microorganisms in the system. The carrier is broadly categorized into two types: biodegradable and nonbiodegradable materials. The common biodegradable carriers are organic polymers such as polycaprolactone and polyacetic acid. The small diameter of 4 mm polycaprolactone carrier was packed in the reactor, which was operated under the nitrogen concentration of 40–50 mg/L and the COD/TN of 3.5–8.0. The nitrogen removal efficiency was around 57–60% [9]. The development of polymer carrier with high porosity was recommended to increase the amount of attached biomass and the reactor performance. The combination of two materials of poly(3-hydroxybutyrate-co-3-hydroxyvalerate) and polyacetic acid was studied in the previous study. The high nitrogen removal of 94% was achieved; however the organic carbon was also observed in the effluent [8]. For the nonbiodegradable carrier, the plastic supporting material was used in an intermittent membrane bioreactor with hydraulic retention time of 22 hours and circle time of 3 hours [10]. Similarly, the commercial plastic material was carrier in the simultaneous partial nitrification, anammox, and denitrification reactor [11].

In this research, two waste materials of corncob and concrete were used as carrier in the attached growth reactor. The corncob which is biowaste represented the biodegradable material and the concrete which is construction waste represented the nonbiodegradable material. The performance of using both carriers on simultaneous nitrification and denitrification was compared. Further, the preliminary study on the attached biomass was also discussed.

## 2. Materials and Methods

### 2.1. Carrier Preparation

The corncob waste was collected from a local market in Phitsanulok, Thailand. The corncob was washed several times to remove the dirt and minerals and then cut into a 2 × 2 × 2 cm size. After that, the corncob carrier was dried at 105°C for two days in a furnace to remove moisture and fungus. Similarly, the concrete waste was collected from a constructed site in the same area. The concrete was washed, cut in a 2 × 2 × 2 cm size, and dried at 105°C for two days. The corncob and concrete carriers were added to the dense activated sludge tanks under continuous air supply for two days. The biomass carriers were transferred to the reactors; the reactor containing corncob carrier was named the corncob reactor and the reactor containing concrete carrier was named the concrete reactor.

### 2.2. Experimental Set-Up and Procedures

**Figure 1:**
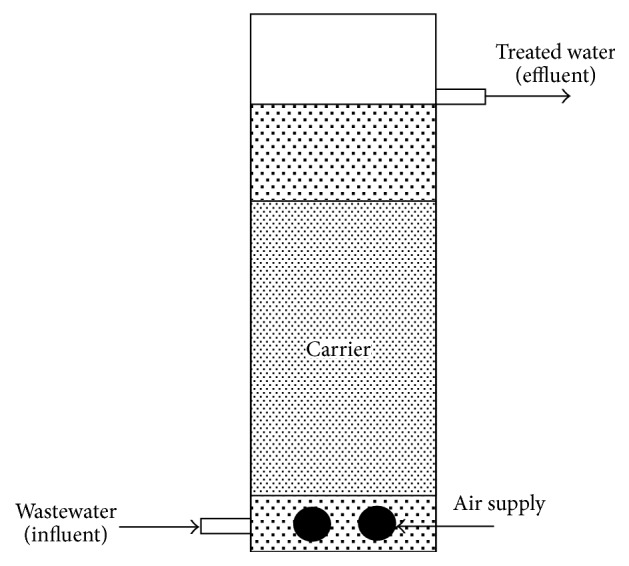
Attached growth reactor set-up.

The schematic of attached growth reactor is shown in Figure 1. The reactor was made of acrylic cylinder with a 11 cm internal diameter and a 3 L working volume. The corncob and concrete carrier were filled up to 60% of the working volume. The synthetic wastewater was continuously fed at the reactor base and the treated water was discharged at the top of the reactor (retention time was approximately 12 hours). The reactor was operated under three hours of aeration and five hours of nonaeration which was suggested in the previous study [12]. The aeration was controlled by suppling the air at the flow rate of 1 L/min via two diffusers; the dissolved oxygen was around 5 mg/L in the aeration. Meanwhile, the air was stopped to supply in the nonaeration period, and the dissolved oxygen was gradually decreasing to 0.5 mg/L by an hour.

The corncob reactor was continuously operated for 25 days under the condition of no external carbon (i.e., acetate) in the influent. On the other hand, the concrete reactor was operated under three phases: no external carbon for 18 days, low carbon (C/N = 2.5) for 30 days, and high carbon (C/N = 3.5) for 30 days (as summarized in Table 1). At the end of operation, the physiological and biochemical characteristics of attached biomass on the corncob and concrete were analyzed.

### 2.3. Batch Test of Carbon Release

Before starting the corncob reactor, the batch test of organic carbon release was prepared. The corncob carrier with no biomass was added in the 2-L distilled water tank, and the ratio of corncob and total volume was 60% which was the same value as in the experiment. The air was continuously supplied to keep the dissolved oxygen at 5 mg/L. The liquid was completely mixed by magnetic stirrer at 200 rpm. The water was sampled and measured the soluble organic carbon for 30 days.

### 2.4. Wastewater Preparation

The synthetic domestic wastewater was used for evaluating the reactor performance. The wastewater was prepared by mixing the following chemicals (g/L): NH_4_Cl 0.15, KH_2_PO_3_ 0.02, MgSO_4_ 0.03, CaCl_2_ 0.36, FeSO_4_ 0.003, and trace element 0.5 mL [13]. The NH_4_-N concentration was controlled at 40 mg/L during the experiment, while NO_2_-N and NO_3_-N were lower than 1 mg/L. For feeding the concrete reactor, the CH_3_COONa 0.34–0.48 g was included in the wastewater.

### 2.5. Analytical Methods

#### 2.5.1. Water Quality

The synthetic wastewater and treated water were sampled for NH_4_-N, NO_2_-N, and NO_3_-N analysis in accordance with the standard method [14]. The NH_4_-N removal efficiency and total nitrogen removal efficiency were calculated as present in (1). The pH, DO, and turbidity were frequently measured using pH meter (Eutech Instruments), DO meter (CyberScan DO 110 Model), and turbidity meter (HACH 2100Q). The organic carbon was measured using TOC analyzer (HACH, IL530 TOC-TN):

(1) NH4-N removal efficiency%=1−NH4effluentNH4influent×100,Total nitrogen removal efficiency%=1−NH4effluent+NO2effluent+NO3effluentNH4influent+NO2influent+NO3influent×100.

#### 2.5.2. Microbial Test

Two samples of biomass were taken from the corncob andconcrete carriers; one was on the carrier surface and another was at the carrier core. The biomass was preliminary study on gram strain, shape, and colony to broadly identify the group of bacteria. Moreover, the same biomass samples were also taken from the unused corncob and concrete carriers to detect the initial bacteria [15].

## 3. Results and Discussion

### 3.1. Performance of Corncob Reactor

**Figure 2:**
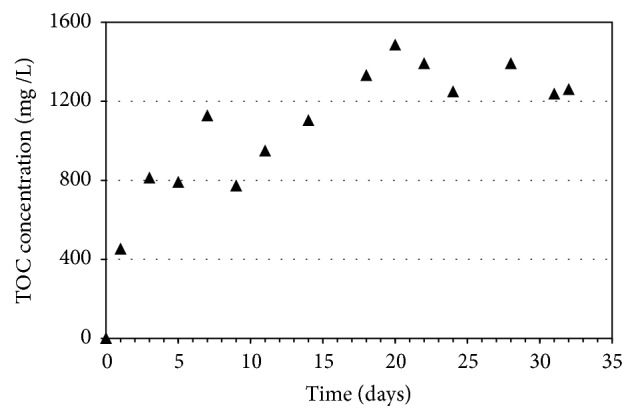
Release of organic carbon from corncob carrier.

Figure 2 shows the release of carbon from corncob carrier during the batch test. The carbon concentration was gradually increased from zero to 810 mg/L at day 3 and continued to 1,120 mg/L at day 7. The amount of soluble carbon reached the maximum of 1,500 mg/L and was stable in the range of 1,200–1,500 mg/L. Moreover, the carbon content in the corncob carrier was also stable during the test (data not shown). According to previous studies [9, 16], the high carbon was continuously released from the biodegradable carrier; however the existence of active biomass was significant factor. Therefore, it can be said that the corncob carrier can be alternative source of carbon for microbial mechanisms in the wastewater treatment system.

**Figure 3:**
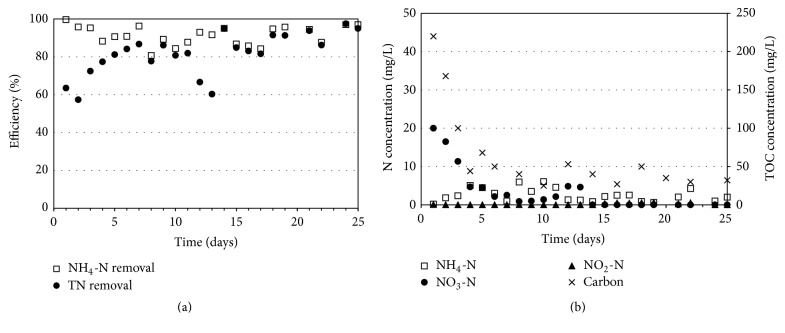
(a) Performance of corncob reactor and (b) effluent concentrations.

When the corncob reactor was started up, the good performance of nitrogen removal from the wastewater was achieved. The efficiency of NH_4_-N removal reached 99% and that of total nitrogen removal was around 63% at the first date as shown in Figure 3(a). After 7 days, the total nitrogen removal efficiency was increased to 87%. Since the acetate was fed as carbon source during the acclimatization, the adaptation period of microorganisms to utilize the corncob carbon was observed in this reactor. As present in Figure 3(b), the high NO_3_-N concentration of 20 mg/L was found in the effluent, even though the high carbon of 220 mg/L remained. It has to be noted that the C/N ratio in the influent is zero because there is no carbon addition in the influent. The key reason that 220 mg/L of carbon concentration was found is the residual carbon from corncob in the reactor. However, the released carbon from the corncob carrier had no effect on the microorganisms activity to oxidize NH_4_-N to NO_3_-N. At the end of experiment, the NH_4_-N and total nitrogen removal efficiencies were constant and greater than 97%.

Figure 3(b) presents that the concentrations of NH_4_-N and NO_2_-N in the effluent were relatively low of 2 and 0.1 mg/L, respectively. Although the NO_3_-N concentration was high in the beginning, the concentration was reduced to 1 mg/L after the adaptation period of 7 days. These results suggested that the nitrification and denitrification simultaneously occurred in the corncob reactor. In addition, the effluent carbon was in the range of 30–50 mg/L, and the turbidity increased from <1 NTU in the influent to 3 NTU in the effluent. This is because the particles from the corncob carrier were released and suspended in the liquid. Although the good nitrogen removal efficiency was found in the corncob reactor, the corncob carrier caused a decreasing quality of treated water from carbon contamination and turbidity.

### 3.2. Performance of Concrete Reactor

**Figure 4:**
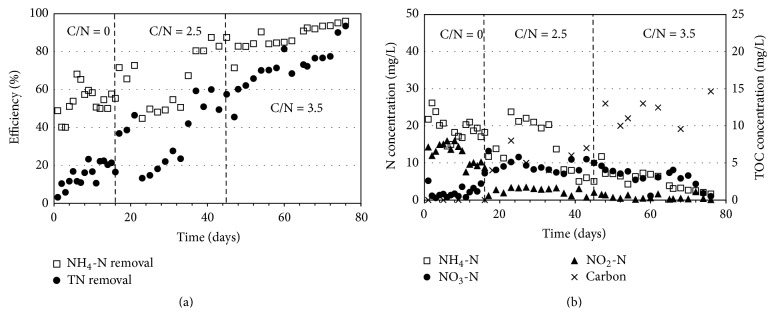
(a) Performance of concrete reactor and (b) effluent concentrations.

Although the concrete is the nonbiodegradable material and does not act as source of carbon for denitrification, the microorganisms were able to utilize the internal carbon from sludge fermentation and intracellular storage (i.e., poly-*β*-hydroxybutyrate, PHA) [17, 18]. Under the no carbon condition, the NH_4_-N concentration was approximately 20 mg/L in the effluent. The low NO_3_-N was observed, while the NO_2_-N concentration was high of 15 mg/L (Figure 4). The results were difference from the corncob reactor which was operated under the same condition and initial biomass. The ineffective nitrification caused the lower attached biomass on the concrete carrier rather than the corncob carrier. According to the surface property and porosity, the corncob carrier was easily attached by active biomass. However, the NH_4_-N and total nitrogen removal efficiency was around 60% and 20% at the end of this phase. It can be seen that the internal carbon was utilized for denitrification; however it was insufficient for complete denitrification. The high NO_2_-N and low carbon in the effluent supported the above explanation. Therefore, the external carbon of acetate was supplied to the reactor in the next experimental phase.

Under the low external carbon of the C/N ratio of 2.5, the NH_4_-N and total nitrogen removal efficiencies increased to 85% and 60%, respectively. This is because the amount of active biomass was increasing by operation time. The low NH_4_-N and NO_2_-N concentrations were detected, while NO_3_-N was suddenly increased to the maximum value of 12 mg/L. The low concentration of carbon in this phase verified that the low C/N ratio of 2.5 was insufficient for complete denitrification. At the high external carbon of the C/N of 3.5, the excellent NH_4_-N and total nitrogen removal efficiencies of 95% were achieved, and all the concentrations of NH_4_-N, NO_2_-N, NO_3_-N, and organic carbon met the effluent standard. In addition, the water quality of effluent was relatively good; the pH was around 7.8 and turbidity was <1 NTU. The concrete was more appropriate carrier than the corncob for biological nitrogen removal at 40 mg/L of NH_4_-N concentration. It has to be noted that the result might be different at other NH_4_-N concentrations because at high NH_4_-N concentration the effluent carbon and the turbidity may be dropped.

### 3.3. Nitrogen Removal Mechanism

**Figure 5:**
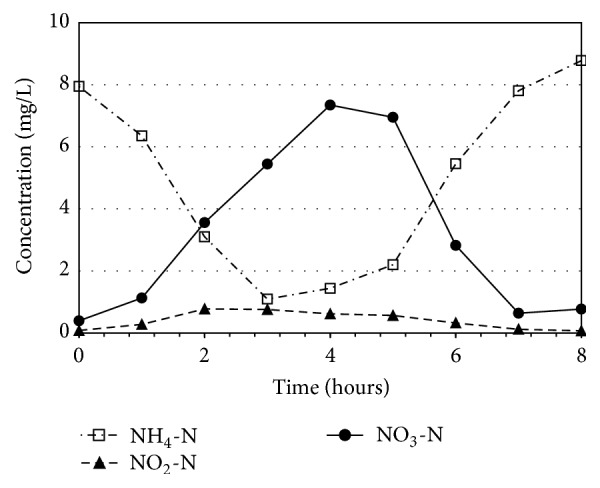
Change of nitrogen concentration in the concrete reactor.

Since the concrete reactor obtained better performance in terms of removal efficiency and water quality, its mechanism of nitrogen removal was identified by collecting the in situ water for eight hours of a cycle period. During continuously feeding the influent NH_4_-N of 40 mg/L, the NH_4_-N concentration was immediately dropped to 8 mg/L, due to the dilution of water in the reactor. In Figure 5, at the three hours of aeration, the NH_4_-N was sharply reduced and changed to NO_3_-N. This phenomenon refers to the fact that the nitrification completely occurred. However, the NO_3_-N concentration kept increasing and reached the highest concentration of 7.3 mg/L at hour 4. This is because the nitrification still occurred using the remaining oxygen. At hours 4 to 8 (nonaeration period), when the DO was nearly zero, the NO_3_-N concentration was decreasing by denitrification and the NH_4_-N was kept to increase. The increase in NH_4_-N concentration was due to the continuous influent feeding and ineffective nitrification occurred. It can be concluded that there were two mechanisms of nitrification and denitrification which occurred in the attached growth reactor; the nitrification mainly occurred in aeration and the denitrification mainly occurred in nonaeration.

### 3.4. Microbial Community

In this research, the physiological and biochemical characteristics including gram stain, shape, and colony were used to broadly categorize the group of bacteria on the carriers. In the corncob reactor, the bacteria on the corncob surface consisted of eight isolates which were categorized into two different groups (see Table 2). Both groups were dominant and also found in the corncob core. However, they were different from the initial bacteria in the unused corncob. These refer to the fact that the corncob contained its own bacteria; however they cannot adapt to the high nitrogen concentration. Thus, other bacteria groups in the initial biomass were grown up and became majority. The bacteria in the carrier surface were responsible for nitrification, while the bacteria at the carrier core acted for denitrification as mentioned in previous study [19].

On the other hand, the characteristics of abundant bacteria on the concrete carrier were the same as found in the corncob carrier. However, the other minor group of bacteria was found at the surface and core. Since no initial bacteria were detected in the unused concrete carrier, the attached bacteria were from the initial biomass. It can be concluded that the bacteria responsible for nitrogen removal in the corncob and concrete reactors were negative gram, cocci shape, positive catalyst test, and smooth and white-turbid colony. However, further microbial analysis is required to identify the bacteria species.

## 4. Conclusion

To utilize the waste material for water treatment, the simple two attached growth reactors were developed to use corncob and concrete as the biomass carrier. The results show that both carriers were good carrier for biomass attachment and the performances were greater than 95% for NH_4_-N and total nitrogen removal. The significant advantage of using corncob carrier was no external carbon. This is because the corncob carrier can release the soluble carbon which was used for denitrification. However, the effluent of corncob reactor contained high turbidity and carbon from corncob degradation. On the other hand, the concrete reactor required the external carbon to achieve high efficiency, in which the minimum value was the C/N ratio of 3.5. In comparison, the concrete was more appropriate carrier than the corncob for nitrogen removal. In addition, the characteristics of abundant bacteria for both carriers were similar which were negative gram, cocci shape, and smooth and white-turbid colony.

## Figures and Tables

**Table 1:** Summary of experimental procedures.

Conditions	Reactor 1	Reactor 2
Carrier	Corncob	Concrete block

External carbon	No	Phase 1: no
		Phase 2: yes (C/N = 2.5)
		Phase 3: yes (C/N = 3.5)

Microbial test	Unused corncob	Unused concrete
	Used corncob (surface)	Used concrete (surface)
	Used corncob (core)	Used concrete (core)

**Table 2:** Physiological and biochemical characteristics of bacteria on corncob and concrete carriers.

Sample	Gram stain	Shape	Catalase test	Characteristics of colony	Color of colony	Number of isolates
Unused corncob carrier	Negative	Rod	−	Smooth	White-turbid	1
	Negative	Rod	+	Smooth	White-turbid	2

Used corncob carrier (surface)	Negative	Cocci	+	Curly	White-turbid	4
	Negative	Cocci	+	Smooth	White-turbid	4

Used corncob carrier (core)	Negative	Cocci	+	Curly	White-turbid	2
	Negative	Cocci	+	Smooth	White-turbid	2

Unused concrete carrier	Not detected	Not detected	Not detected	Not detected	Not detected	Not detected

Used concrete carrier (surface)	Negative	Cocci	+	Smooth	White-turbid	3
	Positive	Rod	+	Smooth	White-turbid	1

Used concrete carrier (core)	Negative	Cocci	+	Smooth	White-turbid	7
	Negative	Cocci	+	Smooth	Brown	1

Symbols: + = positive, − = negative.

## References

[1] Waajen G., Oosterhout F., Douglas G., Lurling M. (2016). Management of eutrophication in Lake De Kuil (The Netherlands) using combined flocculant-Lanthanum modified bentonite treatment. *Water Research*.

[2] Limoges A., de Vernal A., Ruiz-Fernández A.-C. (2015). Investigating the impact of land use and the potential for harmful algal blooms in a tropical lagoon of the Gulf of Mexico. *Estuarine, Coastal and Shelf Science*.

[3] Zhao J., Feng X., Shi X. (2015). Sedimentary organic and inorganic records of eutrophication and hypoxia in and off the Changjiang Estuary over the last century. *Marine Pollution Bulletin*.

[4] Zhang Y., Shi Z., Chen M., Dong X., Zhou J. (2015). Evaluation of simultaneous nitrification and denitrification under controlled conditions by an aerobic denitrifier culture. *Bioresource Technology*.

[5] Ali M., Okabe S. (2015). Anammox-based technologies for nitrogen removal: advances in process start-up and remaining issues. *Chemosphere*.

[6] Leyva-Díaz J. C., López-López C., Martín-Pascual J., Muñío M. M., Poyatos J. M. (2015). Kinetic study of the combined processes of a membrane bioreactor and a hybrid moving bed biofilm reactor-membrane bioreactor with advanced oxidation processes as a post-treatment stage for wastewater treatment. *Chemical Engineering and Processing: Process Intensification*.

[7] Zhao Q., Han H., Hou B., Zhuang H., Jia S., Fang F. (2014). Nitrogen removal from coal gasification wastewater by activated carbon technologies combined with short-cut nitrogen removal process. *Journal of Environmental Sciences*.

[8] Wu W., Yang F., Yang L. (2012). Biological denitrification with a novel biodegradable polymer as carbon source and biofilm carrier. *Bioresource Technology*.

[9] Chu L., Wang J. (2011). Comparison of polyurethane foam and biodegradable polymer as carriers in moving bed biofilm reactor for treating wastewater with a low C/N ratio. *Chemosphere*.

[10] Guo H., Chen J., Li Y., Feng T., Zhang S. (2013). Nitrogen and phosphorus removal in an airlift intermittent circulation membrane bioreactor. *Journal of Environmental Sciences*.

[11] Anjali G., Sabumon P. C. (2015). Development of enhanced SNAD process in a down-flow packed bed reactor for removal of higher concentrations of NH_4_–N and COD. *Journal of Environmental Chemical Engineering*.

[12] Le H. T., Jantarat N., Khanitchaidecha W., Ratananikom K., Nakaruk A. (2015). Development of sequencing batch reactor performance for nitrogen wastewater treatment. *Microbial and Biochemical Technology*.

[13] Guo J., Zhang L., Chen W., Ma F., Liu H., Tian Y. (2013). The regulation and control strategies of a sequencing batch reactor for simultaneous nitrification and denitrification at different temperatures. *Bioresource Technology*.

[14] American Public Health Association (1998). *Standard Methods for the Examination of Water and Wastewater*.

[15] Bergey D. H., John G. H., Noel R. K., Peter H. A. S. (1994). *Bergey's Manual of Determinative Bacteriology*.

[16] Yang X.-L., Jiang Q., Song H.-L., Gu T.-T., Xia M.-Q. (2015). Selection and application of agricultural wastes as solid carbon sources and biofilm carriers in MBR. *Journal of Hazardous Materials*.

[17] Zhang L., Zhang S., Wang S. (2013). Enhanced biological nutrient removal in a simultaneous fermentation, denitrification and phosphate removal reactor using primary sludge as internal carbon source. *Chemosphere*.

[18] Wang X., Wang S., Xue T., Li B., Dai X., Peng Y. (2015). Treating low carbon/nitrogen (C/N) wastewater in simultaneous nitrification-endogenous denitrification and phosphorous removal (SNDPR) systems by strengthening anaerobic intracellular carbon storage. *Water Research*.

[19] Khanitchaidecha W., Nakamura T., Sumino T., Kazama F. (2010). Performance of intermittent aeration reactor on NH_4_-N removal from groundwater resources. *Water Science and Technology*.

